# 2336. General Safety Profile of mRNA-1273 (Moderna COVID-19 Vaccine) in the Elderly Population

**DOI:** 10.1093/ofid/ofad500.1958

**Published:** 2023-11-27

**Authors:** Kate Anteyi, Veronica Urdaneta, Priyadarshani Dharia, Magalie Emile-Backer, Vaishali Khamamkar, Daina Esposito, Walter Straus

**Affiliations:** Moderna, Inc., Cambridge, Massachusetts; Moderna, Inc., Cambridge, Massachusetts; Moderna, Inc., Cambridge, Massachusetts; Moderna, Inc., Cambridge, Massachusetts; Moderna, Inc., Cambridge, Massachusetts; Moderna, Inc., Cambridge, Massachusetts; Moderna, Inc., Cambridge, Massachusetts

## Abstract

**Background:**

Early in the pandemic, the elderly was recognized to be at high risk for COVID-19 related morbidity and mortality and then prioritized for vaccination. To provide further insight supporting the safety of mRNA-1273 in the elderly, we analyzed and characterized adverse events (AE) reported in the Moderna global safety database (GSDB) after mRNA-1273 administration in the elderly population.

**Methods:**

We conducted a descriptive analysis of cumulative data (18 Dec 2020 -17 Dec 2022) from recipients of the mRNA-1273 vaccine, aged >65 years, in the GSDB. Data were aggregated and assessed to search for patterns and variations. Findings were summarized using frequency distributions.

**Results:**

As of 17 Dec 2022, an estimated 773 million doses of mRNA-1273 had been administered, with 658,519 case reports collected in the GSDB. Of these 133,303 cases (20.2%) were in the elderly, 25% were “serious cases”; most reports involved females (65.6%).

Most AE (49.0%) were associated with reactogenicity (including pain, redness, swelling or induration at the injection site, headache, fever, myalgia) similar to results from clinical trials. The most frequently reported events in the elderly were pyrexia, headache, fatigue, chills, and myalgia (Table). Fatal outcomes were reported in 3.3% of the cases. Review of these cases showed they were frequently confounded by common comorbidities of the elderly, including hypertension (42.6%), diabetes mellitus (28.2%), atrial fibrillation (12.6%) and chronic obstructive pulmonary disease (12.2%). These comorbidities provided alternate etiologies for the fatal outcomes.

**Figure:**
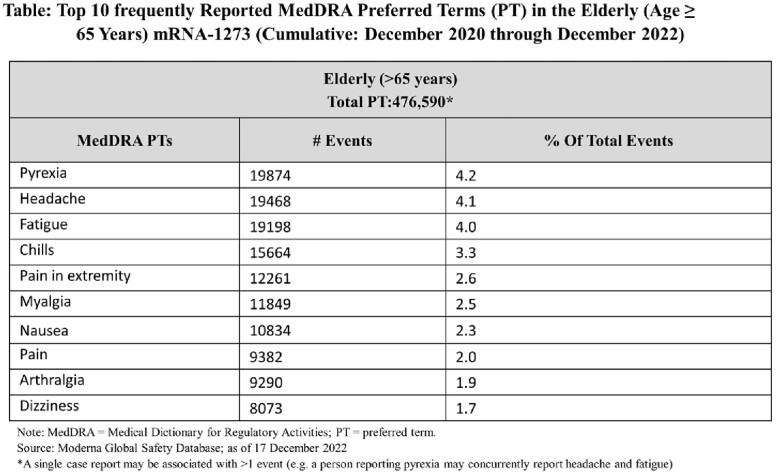

**Conclusion:**

The pattern of adverse events identified in the elderly population was generally consistent with the known safety profile of mRNA-1273 vaccine. This review provides supporting evidence for the favorable safety profile of mRNA-1273 in the elderly population.

**Disclosures:**

**Kate Anteyi, MD, MPH, MBA**, Moderna, Inc.: Salary|Moderna, Inc.: Stocks/Bonds **Veronica Urdaneta, MD, MPH**, Moderna, Inc.: Salary|Moderna, Inc.: Stocks/Bonds **Priyadarshani Dharia, PhD, MD, MPH**, Moderna, Inc.: Salary|Moderna, Inc.: Stocks/Bonds **Magalie Emile-Backer, PharmD, CCRP**, Moderna, Inc.: Salary|Moderna, Inc.: Stocks/Bonds **Vaishali Khamamkar, MS**, Moderna, Inc.: Salary|Moderna, Inc.: Stocks/Bonds **Daina Esposito, PhD, MPH**, Moderna, Inc.: Salary|Moderna, Inc.: Stocks/Bonds **Walter Straus, MD, MPH**, Moderna, Inc.: Salary|Moderna, Inc.: Stocks/Bonds

